# Cerebellar tDCS Effects on Conditioned Eyeblinks using Different Electrode Placements and Stimulation Protocols

**DOI:** 10.3389/fnhum.2017.00023

**Published:** 2017-02-01

**Authors:** Linda Beyer, Giorgi Batsikadze, Dagmar Timmann, Marcus Gerwig

**Affiliations:** Department of Neurology, University of Duisburg-EssenEssen, Germany

**Keywords:** cerebellum, tDCS, eyeblink conditioning, associative learning, extinction

## Abstract

There is good evidence that the human cerebellum is involved in the acquisition and timing of classically conditioned eyeblink responses (CRs). Animal studies suggest that the cerebellum is also important in CR extinction and savings. Cerebellar transcranial direct current stimulation (tDCS) was reported to modulate CR acquisition and timing in a polarity dependent manner. To extent previous findings three experiments were conducted using standard delay eyeblink conditioning. In a between-group design, effects of tDCS were assessed with stimulation over the right cerebellar hemisphere ipsilaterally to the unconditioned stimulus (US). An extracephalic reference electrode was used in Experiment 1 and a cephalic reference in Experiment 2. In both parts the influence on unconditioned eyeblink responses (UR) was investigated by starting stimulation in the second half of the pseudoconditioning phase lasting throughout the first half of paired trials. In a third experiment, effects of cerebellar tDCS during 40 extinction trials were assessed on extinction and reacquisition on the next day. In each experiment, 30 subjects received anodal, cathodal or sham stimulation in a double-blinded fashion. Using the extracephalic reference electrode, no significant effects on CR incidences comparing stimulation groups were observed. Using the cephalic reference anodal as well as cathodal cerebellar tDCS increased CR acquisition compared to sham only on a trend level. Analysis of timing parameters did not reveal significant effects on CR onset and peaktime latencies nor on UR timing. In the third experiment, cerebellar tDCS during extinction trials had no significant effect on extinction and savings on the next day. The present study did not reveal clear polarity dependent effects of cerebellar tDCS on CR acquisition and timing as previously described. Weaker effects may be explained by start of tDCS before the learning phase i.e., offline, individual thresholds and current flow based on individual anatomy may also play role. Likewise cerebellar tDCS during extinction did not modulate extinction or reacquisition. Further studies are needed in larger subject populations to determine parameters of stimulation and learning paradigms yielding robust cerebellar tDCS effects.

## Introduction

During the past two decades transcranial direct current stimulation (tDCS) has been shown to be capable of modulating cortical function. Early studies revealed evidence that cortical excitability was modified in a polarity dependent manner that is it was enhanced by anodal and decreased by cathodal tDCS (Nitsche and Paulus, 2000, 2001). By stimulating cortical cerebral areas, this non-invasive technique has been increasingly used to investigate brain function with various issues (Rogalewski et al., 2004; Antal et al., 2006; Nitsche et al., 2008). In recent years, tDCS has also been applied to the cerebellum to study cerebellar effects on cognitive function and motor learning. Cerebellar tDCS has been shown to modulate working memory, attention and procedural learning (Ferrucci et al., 2008, 2013; Pope and Miall, 2012; Boehringer et al., 2013). Evaluation of the excitability of the primary motor cortex revealed that cerebellar anodal tDCS enhances cerebellar-brain inhibition, whereas cathodal tDCS decreases it (Galea et al., 2009). Anodal tDCS was found to enhance visuomotor adaptation of reaching movements (Galea et al., 2011) and to increase locomotor learning and adaptation (Jayaram et al., 2012; Kaski et al., 2012).

To determine effects of cerebellar stimulation on associative motor learning classical conditioning of the eyeblink reflex appears suitable. Cerebellar dependent delay eyeblink conditioning has been established since decades in animals and humans to study implicit learning processes (for reviews, Thompson et al., 1997; Medina et al., 2002; Timmann et al., 2010; Hesslow et al., 2013). During repeated presentation of an initially neutral conditioned stimulus (CS), commonly a tone, with a coterminating unconditioned stimulus (US), in humans an air puff lateral to the eye or a periorbital electrical shock, subjects learn to lower the eyelid to the tone carefully timed such that this conditioned response (CR) protects the cornea when the air puff arrives. Prior to the acquisition of learned responses a number of CS and US are presented unpaired in a random sequence during the so called pseudoconditioning phase. Following acquisition a series of CS alone trials is presented to extinguish CRs in the extinction phase. There is agreement that the cerebellar cortex as well as the cerebellar nuclei are essentially involved in the acquisition, timing and retention of delay conditioned eyeblink responses (Yeo and Hesslow, 1998; Green and Woodruff-Pak, 2000; Christian and Thompson, 2005; Thompson and Steinmetz, 2009; Boele et al., 2013).

As yet only few studies examined effects of tDCS applied to distinct brain regions on associative learning by using eyeblink conditioning paradigms, in half of them no cerebellar stimulation was performed. In rabbits trace eyeblink conditioning was found modified during tDCS of the somatosensory cortex (Márquez-Ruiz et al., 2012). Acquisition of CRs was significantly enhanced during anodal but reduced during cathodal stimulation suggesting that the sensory perception process in associative learning can be modulated by tDCS. Over the left dorsolateral prefrontal cortex tDCS had no effect on the learning rate or discrimination ratio in a semantic discrimination eyeblink conditioning task (Kotilainen et al., 2015). Finally, delay eyeblink conditioning was used to control for possible effects on cerebellar function in a study investigating the influence of anodal stimulation of the primary motor cortex on locomotor adaptation (Kaski et al., 2012). In that study the reference electrode was placed occipitally in the midline over the inion. However, acquisition and timing of CRs was found unimpaired.

As yet there is one recent study from our group reporting that tDCS applied to the cerebellum can modulate the acquisition and timing of delay conditioned eyeblink responses in a polarity specific manner (Zuchowski et al., 2014). Compared to sham stimulation, anodal tDCS led to improved acquisition whereas this was reduced during cathodal tDCS. Furthermore, timing of CRs was modified. During anodal tDCS CR onset occurred significantly earlier, that is mean onset of responses was shifted closer to the onset of the CS. In that study tDCS was restricted to paired acquisition trials. Across extinction blocks there was no significant difference between stimulation groups in the decline of CRs, although the initial extinction rate appeared to be faster following anodal tDCS.

The aim of the present study was to extent these earlier findings of cerebellar tDCS on the acquisition and timing of conditioned eyeblink responses. At variance with the previous study, tDCS was started “offline” before the beginning of the acquisition phase to assess effects on unconditioned eyeblink responses. In addition, it was of interest whether stimulation during unpaired trials may alter effects of tDCS on acquisition. Furthermore, in order to explore the influence of the position of the reference electrode on behavioral data, two previously reported effective positions were used: the extracephalic reference electrode (Jayaram et al., 2012) in the first and the cephalic reference (Galea et al., 2009) in the second part of the experiment, while keeping the active electrode position constant over the right cerebellum. Using the cephalic electrode, effects on eyeblink conditioning were expected to be more pronounced than using the extracephalic reference. Finally, in a third experiment possible effects of cerebellar tDCS on extinction and savings of conditioned eyeblink responses were investigated. In this experiment tDCS was applied only throughout extinction trials. While extinction may represent an active process of learning, faster extinction during anodal and retardation of extinction during cathodal tDCS was hypothesized.

## Subjects and Methods

Three experiments were performed using delay eyeblink conditioning. In each experiment 30 young and healthy subjects participated that is a total of 90 subjects. In Experiment 1 and 2, tDCS was applied over the right cerebellar hemisphere beginning with the second half of the pseudoconditioning phase and lasting throughout the subsequent first half of the acquisition phase. An extracephalic reference electrode was used in Experiment 1 and a cephalic reference in Experiment 2. In each of the two experiments, CR incidences as a measure of learning were compared between stimulation groups (anodal vs. cathodal vs. sham). In addition, CR timing was analyzed across blocks and between groups. Furthermore, timing of unpaired trials at the beginning of each experiment with and without stimulation was compared within Experiments 1 and 2. Finally, in each experiment extinction was analyzed between stimulation groups.

In participants of Experiment 3 no stimulation was given during the acquisition phase, cerebellar tDCS was applied only during the extinction phase using the cephalic reference. In addition saving effects were tested without stimulation on the next day. Again, CR incidences were compared between stimulation groups for acquisition and extinction on the first day and the reacquisition phase on the next day. In each of the three experiments subjects received anodal, cathodal or sham stimulation in a double-blinded fashion (10 subjects randomly allocated to the stimulation subgroups).

### Subjects

Out of the 90 healthy individuals 45 were female, 45 male, the mean age in Experiment 1 was 23.5 ± 2.3 years, in Experiment 2 23.6 ± 1.7 years and in Experiment 3 23.3 ± 2.8 years. None of the subjects had a history of neurological diseases or used centrally acting substances; the clinical examination did not reveal cerebellar or other neurological signs. All subjects were naïve to eyeblink conditioning and tDCS. In each subject hearing thresholds (dB SPL) were determined using a tone of 1 KHz, values were within normal age limits in all participants. The local ethics committee of the University of Duisburg-Essen approved the study and written informed consent was obtained from all subjects in accordance with the Declaration of Helsinki.

### Experimental Procedures

#### Experiment 1 and 2

In both experiments all groups experienced the same eyeblink conditioning protocol. Cerebellar tDCS was applied over the right cerebellar hemisphere. In Experiment 1 the deltoid muscle was used as an extracephalic reference and in Experiment 2 the buccinator muscle as a cephalic reference.

#### Eyeblink Conditioning

According to Gormezano and Kehoe (1975) a standard delay eyeblink conditioning protocol was used, for further details see Gerwig et al. (2003, 2005). Subjects were seated in a chair, both arms lying comfortably on armrests. To maintain vigilance and attention a silent movie was shown on a screen, positioned in a distance of 2 m to the subject. The CS consisted of an initially neutral tone (1 KHz, 70 dB SPL, duration 540 ms) provided via headphones and superimposed on a continuous white noise of 60 dB SPL to mask environmental noise. The CS was given ipsilaterally to the US, an air puff (duration 100 ms, intensity 400 KPa at source, 110 KPa at nozzle) directed laterally to the outer canthus of the right eye through a nozzle mounted on a helmet which was worn by the subject. The air puff was followed by a reflexive blink, the unconditioned response (UR).

**Figure 1:**
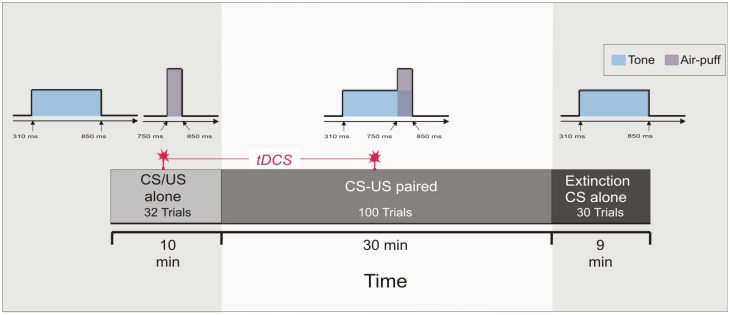
**Experimental protocol: Experiment 1 and 2**. At the beginning 16 conditioned stimulus (CS) alone trials and 16 unconditioned stimulus (US) alone trials were presented in a random sequence (pseudoconditioning), followed by 100 paired CS-US trials. Thirty CS alone extinction trials were given at the end. Transcranial direct current stimulation (tDCS) was started after 16 pseudoconditioning trials and lasted throughout the first 50 paired trials of the acquisition phase, i.e., across 20 min.

**Figure 2:**
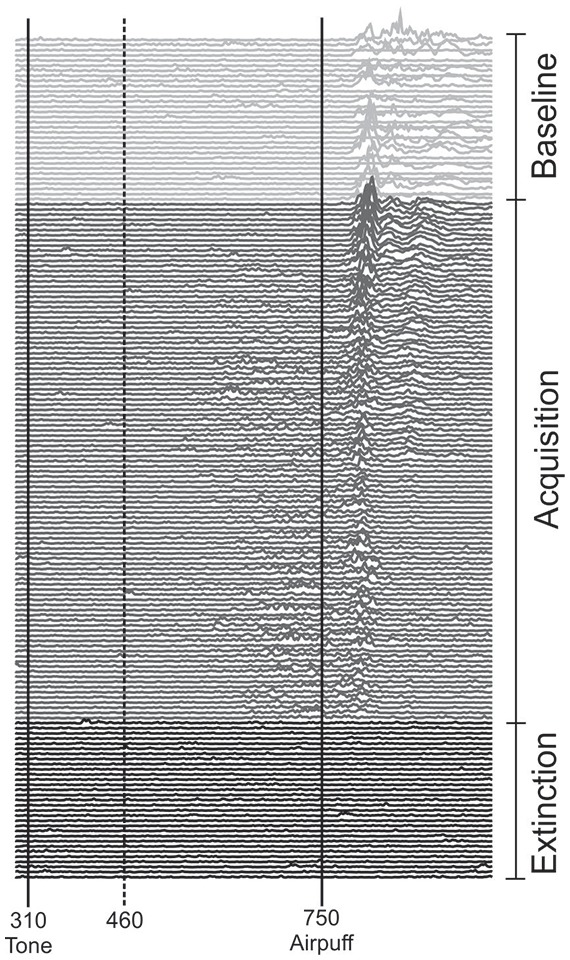
**Eyeblink conditioning in an individual subject during anodal tDCS using the cephalic reference electrode**. Rectified and filtered EMG-data of the orbicularis oculi muscle are shown from the beginning with the unpaired phase (top) consisting of 16 CS alone and 16 US alone trials in a random sequence followed by 100 CS-US paired trials and 30 CS alone extinction trials at the end of the experiment (bottom). tDCS was started with the second half of unpaired and lasted throughout the first half of paired trials. The vertical lines indicate the onset of the CS and US. Responses occurring within the 150 ms interval after CS onset (dotted line) were considered alpha-responses. See “Subjects and Methods” Section for further details.

Each of the two experiments started with a pseudoconditioning phase consisting of 16 CS alone and 16 US alone trials presented in a random sequence. This was followed by the acquisition phase of 100 CS-US paired trials and 30 CS alone extinction trials at the end (Figure 1). The intertrial interval varied randomly between 16, 18 and 20 s (randomized within each subject, but not between subjects). In paired trials the CS started 310 ms after onset of each trial, preceded the US onset by a fixed time interval of 440 ms and coterminated with the US. Responses were recorded from orbicularis oculi muscles bilaterally using electromyographic surface electrodes fixed to the lower eyelid and to the nasion. The grounding electrodes were placed on the subjects’ forearm. Signals were fed to EMG amplifiers (sampling rate 1000 Hz, band pass filter frequency between 100 Hz and 2 kHz), full wave-rectified and further low pass-filtered offline (100 Hz). An individual example of recorded eyeblink conditioning traces during anodal tDCS using the cephalic reference electrode is given in Figure 2.

In paired and extinction trials conditioned eyeblink responses (CR) were semiautomatically identified using custom made software (see Gerwig et al., 2010). Rectified EMG recordings were filtered using a series of non-linear Gaussian filters. In each recording with a minimum duration of 20 ms and a minimum integral of 1 mV*ms, response onset was defined at the time point where EMG activity reached 7.5% of the EMG maximum. Trials were visually inspected and implausible identification of CRs was manually corrected. Responses occurring within the 150 ms interval after CS onset were considered as reflexive responses to the tone (i.e., alpha-responses) and not CRs (Woodruff-Pak et al., 1996). Trials with spontaneous blinks occurring prior to CS onset were excluded from the analysis (Bracha et al., 2000). The total number of paired trials was subdivided into blocks of 10 trials each. Accordingly, CS alone extinction trials were subdivided in blocks of 10 trials each.

The number of CRs was expressed as the percentage of trials containing responses with respect to each block of 10 trials (percentage CR incidence) and the total number of trials (total percentage CR incidence). Timing parameters of CRs in paired trials, and URs in unpaired trials were semiautomatically quantified as outlined above. CR onset and peaktime were expressed as negative values prior US onset set as 0 ms. Individual values for CR and UR onset and time to peak were averaged. CR timing was analyzed only in those subjects who exhibited CRs in each block. EMG amplitudes were not analyzed due to methodological considerations in surface EMG recordings, e.g., individual differences in skin properties (Gerwig et al., 2007). The frequency of spontaneous blinks was measured in each session within 1 min both at the beginning and the end of the experiment. The rate of alpha-blinks was analyzed within the 150 ms interval after CS onset of 100 paired trials.

#### transcranial Direct Current Stimulation (tDCS)

In both Experiment 1 and 2, cerebellar tDCS was applied through two electrodes with a surface area of 35 cm^2^ (5 cm × 7 cm, vertical orientation) connected to a neuroConn DC-Stimulator Plus (serial number 0371; neuroConn GmbH). A special electrode paste was used. The stimulating electrode was centered 3 cm lateral to the inion over the right cerebellar hemisphere. The reference electrode was placed in a vertical position on the ipsilateral deltoid muscle in Experiment 1 and on the buccinator muscle in Experiment 2 (Galea et al., 2009, 2011; Jayaram et al., 2012). The maximum current was set to 2 mA (Iyer et al., 2005) with a ramp-like fade-in and fade-out of 30 s (current density 0.057 mA/cm^2^). The stimulation was started after the first 16 of the CS and US alone trials that is after the first half of the pseudoconditioning phase and lasted throughout 50 of the paired CS-US trials of the acquisition phase (Figure 1). The overall duration of stimulation in each experiment was 20 min. The fade-in-short stimulation-fade out stimulation protocol was used in the sham session. The current was ramped up for 20 s, followed by 40 s of tDCS and then ramped down for 10 s. Because of higher impedance levels in Experiment 3 the fade-in and fade-out was reduced to 10 s. The neuroConn DC-Stimulator Plus was programmed with three tDCS settings (anodal, cathodal and sham stimulation). Randomly a defined setting was chosen and anodal, cathodal or sham tDCS was applied. The modality of stimulation was unknown to the participant and investigator.

#### Experiment 3

In this part of the study cerebellar tDCS was restricted to the extinction phase using the cephalic reference over the buccinator muscle ipsilateral to the stimulation. Saving effects were investigated on the next day. Eyeblink conditioning was modified as follows.

#### Eyeblink Conditioning

**Figure 3:**
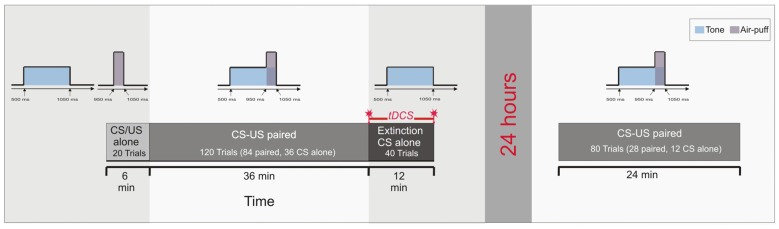
**Experimental protocol: Experiment 3**. Ten CS alone trials and 10 US alone trials were presented at the beginning in a random sequence (pseudoconditioning), followed by the acquisition phase of 84 paired CS-US trials and 36 CS alone trials interspersed. At the end 40 CS alone trials were given as extinction trials. tDCS was applied throughout extinction trials lasting for 12 min. Saving effects were tested on the next day 24 h later using 28 paired CS-US trials and 12 CS alone trials interspersed.

The pseudoconditioning phase consisted of 10 CS alone and 10 US alone trials in an unpaired and random sequence as outlined above. In paired trials of Experiment 3 the CS started 500 ms after onset of each trial and lasted for 550 ms coterminating with the US of 100 ms duration. In the acquisition phase 84 CS-US paired trials were presented with 36 CS alone trials being interspersed followed by the extinction phase of 40 CS alone trials (Figure 3). In the reacquisition phase on the next day 56 CS-US paired trials with 24 CS alone trials interspersed were presented. In the acquisition and reacquisition phases CS alone trials were randomized within each subject, but not between subjects. CS alone trials were followed by one, two, three or five CS-US paired trials, respectively (mean 2.2 trials). The intertrial interval varied between 16, 18 and 20 s.

#### transcranial Direct Current Stimulation (tDCS)

In Experiment 3, cerebellar tDCS was performed only throughout the 40 extinction trials lasting for 12 min and using the cephalic reference. There was no stimulation during the reacquisition on the next day (Figure 3). Technical details of stimulation, the montage of electrodes, maximum current and current density were the same as outlined above. Again anodal, cathodal and sham stimulation were applied in a double-blinded fashion.

#### Data Analysis

In Experiment 1 and 2 analysis of variance (ANOVA) with percentage CR incidence as dependent variable, block (1–10: 10 blocks of 10 paired trials) as within subject factor and stimulation group (anodal vs. cathodal vs. sham) as between subject factor was calculated. CR timing was analyzed by ANOVA with mean CR onset and peaktime as dependent variable and stimulation group as between subject factor. Mean values of blocks of 20 paired trials were used. CR timing was analyzed in those subjects who showed CRs in each block. Accordingly, ANOVA was calculated in extinction trials in Experiment 1 and 2 comparing CR incidences in block 10 of paired trials with extinction block 3. Unconditioned eyeblink responses were compared between stimulation groups using one-way ANOVA. In each group UR timing was compared between responses with and without stimulation. Rates of spontaneous blinks and alpha blinks were compared between stimulation groups using one-way ANOVA. In Experiment 3 ANOVA with repeated measures was calculated in paired trials and interspersed CS alone trials with percentage CR incidence as dependent variable, block (1–12 containing on average seven paired trials and three CS alone trials per block) as within subject factor and (later) stimulation group (anodal vs. cathodal vs. sham) as between subject factor. Extinction effects during stimulation were determined by the decline of CRs from the last block of paired trials across the four extinction blocks compared between stimulation groups as between subject factor. In addition, CR timing in extinction trials was compared between groups. To determine savings ANOVA which was calculated comparing the acquisition phase on the first day and the eight reacquisition blocks on the second day with block and phase (acquisition vs. reacquisition) as within subjects factors and stimulation group as between subjects factor. Level of significance was set at *p* < 0.05. For all effects, the degrees of freedom were adjusted, if appropriate, according to Greenhouse and Geisser.

## Results

Participants tolerated cerebellar tDCS well. A mild tingling was reported at the beginning of the stimulation beneath the electrodes, this disappeared after several minutes in most of the participants. No other side effects occurred.

### Experiment 1 and 2

#### CR Acquisition

**Figure 4:**
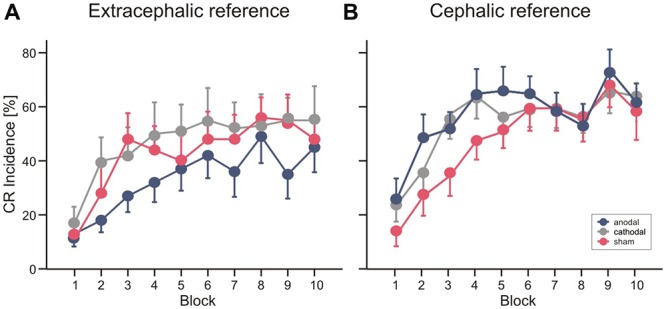
**Experiment 1 using an extracephalic reference** **(A)** and Experiment 2 using a cephalic reference **(B)**. Mean percentage conditioned response (CR) incidence and standard errors (SE) in paired trials shown per block of 10 trials during anodal (blue circles), sham (red circles) and cathodal tDCS (gray circles). See “Results” Section for further details.

Mean percentage of CR incidences standard errors (SE) across the 10 blocks of paired trials in the different stimulation modalities are shown in Figure 4. In Experiment 1 ANOVA with percentage of CR incidence as dependent variable, block (1–10) as the within subject factor and stimulation group (anodal vs. cathodal vs. sham) as the between subject factor did not reveal a significant main effect of group (*F*_(2,27)_ = 0.8; *p* = 0.45). The block effect was significant (*F*_(1,27)_ = 16.0; *p* < 0.001), the block by group interaction effect was not significant (*F*_(2,27)_ = 0.8; *p* = 0.62). Also the comparison between anodal or cathodal vs. sham and anodal vs. cathodal stimulation did not show significant block by group and group effects (all *p* values > 0.2). During anodal tDCS, using the extracephalic reference, percentage of CR incidence increased from 13.0 ± 4.7% in block 1 to a maximum of 49.0 ± 9.8% in block 8 (mean total CR incidence 33.8 ± 18.1%). In the sham stimulated group there was a larger increase across blocks from 11.0 ± 5.0% in block 1 to a peak of 56.0 ± 7.5% in block 8 (mean total CR incidence 42.6 ± 25.1%). Mean total percentage CR incidence was highest in the cathodal stimulated group (47.0 ± 28.2%) with an increase from 17.0 ± 6.0% in block 1 to a peak of 55.0 ± 12.3% in block 10.

In Experiment 2 with the cephalic reference over the ipsilateral buccal muscle, ANOVA with percentage of CR incidence as dependent variable, block (1–10) as the within subject factor and stimulation group as the between subject factor did not reveal a significant main effect of group (*F*_(2,27)_ = 2.3; *p* = 0.11). The block effect was significant (*F*_(1,27)_ = 25.3; *p* < 0.001), the block by group interaction effect was not significant (*F*_(2,27)_ = 0.6; *p* = 0.75). Comparing the anodal and sham stimulated group showed a main effect of group close to significance (*F*_(1,18)_ = 4.3; *p* = 0.051). The comparison of cathodal vs. sham stimulation (*F*_(1,18)_ = 3.2; *p* = 0.09) and anodal vs. cathodal stimulation (*F*_(1,18)_ = 0.2; *p* = 0.67) did not reveal significant group effects. Percentage of CR incidence with anodal stimulation increased from 24.0 ± 7.0% in block 1 to a peak of 68.0 ± 7.1% in block 9 (mean total CR incidence 52.6 ± 18.2%). In the sham stimulated group the increase across blocks was from 12.0 ± 4.2% in block 1 to 63.0 ± 7.6% in block 9 (mean total CR incidence 43.8 ± 16.2%) and in the cathodal group from 22.0 ± 5.7% in block 1 to a peak of 61.0 ± 7.7% in block 9 (mean total percentage CR incidence 50.2 ± 14.1%).

Finally, a direct comparison between groups was performed including all subjects of Experiment 1 and 2 using the cephalic as well as the extracephalic reference electrode. ANOVA with percentage of CR incidence as dependent variable, block (1–10) as the within subject factor and reference electrode (extracephal vs. cephal) and stimulation group (anodal vs. cathodal vs. sham) as the between subject factor did not reveal a significant main effect neither of reference electrode (*F*_(1,54)_ = 2.2; *p* = 0.14) nor of stimulation modality (*F*_(2,54)_ = 0.48; *p* = 0.62) nor significant interaction effects (all *p* values > 0.2).

#### Timing of Conditioned and Unconditioned Eyeblink Responses

ANOVA was calculated with CR onset and peaktime as dependent variables, block as within and stimulation group as between subject factor. Group effects were not significant for CR onset (*F*_(2,26)_ = 1.4; *p* = 0.26) and not for CR peaktime (*F*_(2,26)_ = 1.4; *p* = 0.27). Analysis included 10 subjects in each, the anodal and sham group and nine subjects in the cathodal stimulated group. ANOVA did not reveal a significant group effect neither for CR onset (*F*_(2,22)_ = 0.1; *p* = 0.88) nor for CR peaktime (*F*_(2,22)_ = 0.001; *p* = 0.99). For analysis of timing data, values for CR onset and time to peak were averaged across 20 paired trials and compared across five blocks à 20 trials. In Experiment 1 using the extracephalic reference electrode analysis of CR timing included nine subjects in the anodal and eight subjects in each, the cathodal and sham stimulated group. Mean values of CR onset and peaktime latencies across paired trials in Experiment 1 and 2 are presented in Table 1.

**Table 1:** **Means and standard deviations (SD) of onset and peaktime latencies of conditioned (CRs) and unconditioned eyeblink responses (URs) in paired trials, unpaired trials in each experiment and conditioned stimulus (CS) only trials on the first (D1) and second day (D2) in Experiment 3 in the three stimulation modalities**.

	Anodal	Cathodal	Sham
**Experiment 1**
*CR onset*	−131.1 ± 26.2	−142.0 ± 32.2	−143.6 ± 28.5
*CR peaktime*	−97.3 ± 29.9	−104.4 ± 28.8	−113.1 ± 31.2
*UR onset*	45.4 ± 04.5	44.4 ± 05.7	42.3 ± 04.7
*UR peaktime*	95.9 ± 14.8	100.5 ± 11.4	81.5 ± 31.8
UR onset*	44.4 ± 05.2	43.1 ± 04.9	43.6 ± 04.2
UR peaktime*	85.1 ± 06.6	87.6 ± 09.8	76.6 ± 27.9
**Experiment 2**
*CR onset*	−144.5 ± 34.8	−152.0 ± 32.4	−131.1 ± 20.9
*CR peaktime*	−126.4 ± 34.7	−133.3 ± 27.0	−113.1 ± 23.3
*UR onset*	42.8 ± 03.5	42.9 ± 07.1	42.2 ± 04.6
*UR peaktime*	90.1 ± 12.4	92.8 ± 11.6	87.2 ± 07.4
UR onset*	44.2 ± 04.1	42.3 ± 03.3	41.8 ± 05.3
UR peaktime*	77.2 ± 06.3	79.7 ± 09.5	85.5 ± 10.5
**Experiment 3**
*CR onset D1 (paired)*	−160.9 ± 20.4	−163.9 ± 12.2	−151.7 ± 11.1
*CR peaktime D1 (paired)*	−140.2 ± 17.5	−142.6 ± 12.7	−128.8 ± 09.9
*CR onset D1 (CSonly)*	−152.7 ± 31.8	−138.7 ± 24.2	−146.9 ± 32.4
*CR peaktime D1 (CSonly)*	−128.3 ± 31.5	−111.3 ± 19.2	−117.6 ± 32.7
*Exinction onset*	−162.0 ± 54.4	−174.1 ± 45.5	−178.3 ± 33.3
*Extinction peaktime*	−110.1 ± 54.0	−130.7 ± 55.1	−130.3 ± 32.7
*CR onset D2 (paired)*	−155.4 ± 30.7	−145.7 ± 35.9	−143.4 ± 29.1
*CR peaktime D2 (paired)*	−125.4 ± 26.4	−115.0 ± 36.8	−112.4 ± 25.3
*CR onset D2 (CSonly)*	−153.3 ± 40.2	−140.4 ± 49.7	−162.1 ± 41.0
*CR peaktime D2 (CSonly)*	−122.5 ± 39.8	−109.2 ± 46.1	−133.3 ± 40.4
*UR onset*	38.1 ± 05.5	70.2 ± 95.0	39.2 ± 07.6
*UR peaktime*	93.9 ± 13.6	125.1 ± 91.7	103.0 ± 18.2

Note that (negative) values for CR onset and peaktime latencies refer to the time prior to the onset of the US (air puff), set as 0 ms. The number of included subjects varies because of missing CRs in individual blocks. *Indicates URs in the second half of the pseudoconditioning phase with tDCS.

In unpaired trials ANOVA was calculated with UR onset and peaktime as dependent variables, with and without tDCS as within and stimulation group as between subject factor. No significant group effects were observed in Experiment 1 for mean UR onset (*F*_(2,27)_ = 0.5; *p* = 0.60) and mean UR peaktime (*F*_(2,27)_ = 1.7; *p* = 0.19) and in Experiment 2, respectively (UR onset: *F*_(2,27)_ = 0.3; *p* = 0.72; mean UR peaktime: *F*_(2,27)_ = 0.4; *p* = 0.69). Note that the first 16 URs were given before and the second half after start of tDCS (anodal vs. cathodal vs. sham; for mean values see Table 1).

#### Extinction

In Experiment 1 ANOVA comparing block 10 of paired trials vs. extinction block 3 revealed a significant extinction effect (*F*_(1,27)_ = 38.8; *p* < 0.001). The extinction by group (*F*_(2,27)_ = 0.07; *p* = 0.93) and the group effect (*F*_(2,27)_ = 0.86; *p* = 0.43) were not significant. CR incidences decreased across the three extinction blocks in the anodal and sham stimulated groups, the decline was less in the cathodal group. In the third extinction block mean CR incidences were: anodal 11.0 ± 5.0%, cathodal 23.0 ± 4.2%, sham 11.0 ± 5.8%. In Experiment 2 ANOVA revealed a significant extinction effect (*F*_(1,27)_ = 89.2; *p* < 0.001) comparing block 10 of paired trials vs. extinction block 3. The extinction by group (*F*_(2,27)_ = 0.64; *p* = 0.53) and the group effect (*F*_(2,27)_ = 0.52; *p* = 0.60) were not significant. There was also a decline of CR incidences across extinction blocks in the three stimulation groups. In the third extinction block mean CR incidence were: anodal 6.0 ± 2.7%, cathodal 12.0 ± 7.8%, sham 16.0 ± 5.8%.

#### Spontaneous Blink-Rate and Alpha-Blinks

In Experiment 1 the mean number of spontaneous blinks was not different between the stimulation groups as revealed by one-way ANOVA (at the beginning: *F*_(2,27)_ = 0.21; *p* = 0.81; at the end: *F*_(2,27)_ = 0.64; *p* = 0.53); (anodal: at the beginning 20.2 ± 2.9 blinks/min, at the end 15.6 ± 2.0; cathodal: at the beginning 22.5 ± 3.2 blinks/min, at the end 19.3 ± 2.6; sham: at the beginning 22.4 ± 2.4 blinks/min, at the end 20.0 ± 3.9). This was also the case in Experiment 2 as revealed by ANOVA (at the beginning: *F*_(2,27)_ = 1.4; *p* = 0.26; at the end: *F*_(2,27)_ = 0.06; *p* = 0.94); (anodal: at the beginning 27.1 ± 6.2 blinks/min, at the end 16.6 ± 4.0; cathodal: at the beginning 17.6 ± 2.4 blinks/min, at the end 18.2 ± 3.1; sham: at the beginning 23.1 ± 1.8 blinks/min, at the end 17.3 ± 2.1). In Experiment 1 one-way ANOVA revealed significantly different alpha-blinks between groups (*F*_(2,27)_ = 4.3; *p* = 0.023) based on enhanced alpha-blinks in the cathodal stimulated group. Alpha-blinks did not differ between groups in Experiment 2 (*F*_(2,27)_ = 0.6; *p* = 0.57).

### Experiment 3

**Figure 5:**
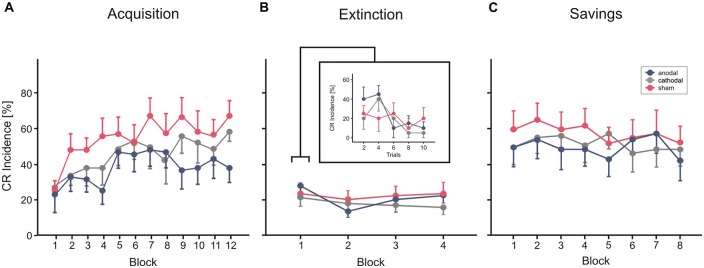
**Experiment 3 using a cephalic reference**. Mean percentage CR incidence and SE in acquisition trials **(A)**, extinction **(B)** and savings on the next day **(C)**, shown per block of 10 trials. Anodal (blue circles), sham (red circles) and cathodal tDCS (gray circles) was applied during extinction trials. CR incidences in block 1 of extinction trials are shown more detailed in blocks of two trials. Decline of CRs across the four extinction blocks was not significantly different comparing stimulation groups. See “Results” Section for further details.

Figure 5 shows mean CR incidences in the acquisition and extinction of CRs as well as savings on the following day in the three stimulation groups.

#### CR Acquisition

In each of the three groups mean percentage of CR incidences increased across the 10 blocks of paired trials (Figure 5A). Note that no stimulation was applied during acquisition in this part of the study. ANOVA with percentage of CR incidence as dependent variable, block (1–12) as the within subject factor and group as the between subject factor did not reveal a significant main effect of group (*F*_(2,27)_ = 1.3; *p* = 0.28). The block effect was significant (*F*_(1,27)_ = 6.6; *p* < 0.001), the block by group interaction effect was not significant (*F*_(2,27)_ = 0.9; *p* = 0.57). Mean total CR incidence, comprising CRs of the paired and CS only trials, was 37.2 ± 22.0%, 44.3 ± 22.3% and 53.2 ± 22.0% in the thereafter anodal, cathodal and sham stimulated subjects.

#### Extinction

Each of the stimulation groups reduced CR incidences during tDCS in extinction trials. ANOVA with repeated measures was conducted with block (*n* = 5, including the last acquisition block and the four extinction blocks) as within subjects factor, and group as between subjects factor. The block (that is extinction) effect was significant (*F*_(1,27)_ = 0.6, *p* < 0.001), the extinction by group interaction effect tended to significance (*F*_(2,27)_ = 0.3; *p* = 0.051), the group effect was not significant (*F*_(2,27)_ = 1.0; *p* = 0.38). To consider extinction in more detail, the 10 trials of the first extinction block were analyzed separately in blocks by two trials (Figure 4B). ANOVA revealed a significant extinction effect (*F*_(1,27)_ = 3.8; *p* = 0.014), the block by group (*F*_(2,27)_ = 1.0; *p* = 0.41) and the group effect (*F*_(2,27)_ = 0.4; *p* = 0.65) were not significant. A numerical reduction was observed in the first extinction block compared to block 12 of the acquisition trials in all groups (anodal: 31.0 ± 20.2% in block 12 to 24.0 ± 11.7% in Ext 1, cathodal: 47.0 ± 13.4% block 12 to 18.0 ± 14.0% in Ext 1, sham: 54.0 ± 21.2% in block 12 to 20.0 ± 17.6% in Ext 1). Analysis of timing parameters in extinction trials did not reveal significant group and group by stimulation effects for CR onset and CR peaktime (all *p* values > 0.4 and > 0.5, respectively).

#### Savings

Subjects of all three stimulation groups showed saving effects on the next day (Figure 5C). Comparing mean CR incidences between groups ANOVA did not reveal significant block, block by group and group effects (all *p* values > 0.3). However, there was a higher mean CR incidence in the first reacquisition block compared to the first, but similar to the last acquisition block (mean CR incidence reacquisition block 1: 44.0 ± 31.0%, 44.0 ± 27.6% and 53.0 ± 30.2% in the—during extinction trials—anodal, cathodal and sham stimulated subjects). ANOVA with block (*n* = 8, that is, the first eight blocks in acquisition and the eight reacquisition blocks) and phase (acquisition vs. reacquisition) as within subjects factors and stimulation group as between subjects factor, revealed a significant effect of phase, that is day (*F*_(1,27)_ = 22.3; *p* < 0.001) indicating higher savings than acquisition, and significant phase by block interaction effect (*F*_(1,27)_ = 5.0; *p* < 0.001). The phase by group effect (*F*_(2,27)_ = 0.2; *p* = 0.85) and the group effect (*F*_(2,27)_ = 0.5; *p* = 0.60) were not significant.

## Discussion

In the present study, anodal as well as cathodal cerebellar tDCS led to a modulation of CR acquisition at a trend level compared to sham when a cephalic reference electrode was used. This was not observed using an extracephalic reference, there were no significant group differences of mean CR incidences comparing stimulation groups. Analysis of timing parameters did not result in significant effects of cerebellar tDCS on CR onset and peaktime latencies in both experiments. In the third experiment, cerebellar tDCS during extinction trials had no significant effect on extinction and savings on the next day.

Overall, the first two experiments of the present study did not reveal clear polarity dependent effects of cerebellar tDCS on the acquisition of conditioned eyeblink responses as previously described. In the study by Zuchowski et al. (2014) the acquisition of CRs was significantly enhanced during anodal cerebellar tDCS but was markedly reduced by cathodal stimulation compared to the sham condition. These findings were consistent with results of adaptive motor learning tasks (Galea et al., 2011; Jayaram et al., 2012; Kaski et al., 2012).

In the second experiment of the present study the identical montage of electrodes was used with the cephalic reference electrode fixed to the buccinator muscle ipsilaterally. In some contrast to our previous findings, CR incidences showed only a tendency to increase during anodal but also during cathodal stimulation. No significant group effects were found using the extracephalic reference over the deltoid muscle.

There may be several reasons why the present experiments did not yield obvious effects on CR acquisition as previously reported. Different to our previous study, tDCS was started before the learning phase that is within the second half of unpaired pseudoconditioning trials to detect possible effects of stimulation on unconditioned eyeblink responses. Some animal studies suggest that the cerebellum is less involved in learning *per se* but mainly in the performance of learned responses (Gruart et al., 2000; Delgado-García and Gruart, 2002; Seidler et al., 2002; Jiménez-Díaz et al., 2004). However, no significant effects of stimulation on UR timing were observed in the present subjects. This is consistent with data of a theta-burst stimulation study in a group of patients with dystonia who were stimulated before eyeblink conditioning (Hoffland et al., 2013). The present findings suggest that stimulation affects primarily CR acquisition, as shown in the previous study by Zuchowski et al. (2014) and not the performance of eyeblink responses in general. An effect on the performance of CRs, however, cannot be excluded by the present data. There is evidence from other brain regions that behavioral effects of tDCS appear to depend on the relative timing of the stimulation and the task execution. Concurrent anodal tDCS and performance of an implicit learning task led to an improved rate of learning (Nitsche et al., 2003; Reis et al., 2009). However, when the task was performed after a period of stimulation, the learning rate was reported to be unchanged (Kuo et al., 2008). Moreover, tDCS of the primary motor cortex during a sequence-learning task modulated learning rates in a polarity-specific manner (Stagg et al., 2011). When applied prior to the task anodal as well as cathodal tDCS was followed by a slowing of learning compared to sham stimulation. Metaplastic mechanisms are discussed to explain these interactions. Likewise, cerebellar tDCS may change synaptic plasticity within cerebellar circuitry and stimulating during the unpaired session could lead on to masking effects on acquisition. That is application of tDCS already during unpaired trials may enhance circuitry to represent the CS and US alone and not the link between the two stimuli. On the other hand, starting tDCS with the presentation of paired CS-US trials could enhance those circuits promoting the linking process. However, this interpretation is very hypothetical and requires future experiments addressing this particular issue.

No significant group effects on CR incidences were observed when the extracephalic reference electrode attached to the ipsilateral arm was used. Even following anodal tDCS mean CR incidences remained lower compared with cathodal or sham stimulation. However, although direct comparison between Experiment 1 and 2 did not reveal significant effects, potential differences in the use of extracephalic and cephalic reference electrodes are shortly discussed. Using computational electromagnetic techniques to evaluate the electric field and current density induced by cerebellar tDCS Parazzini et al. (2014) reported that cerebellar tDCS with an extracephalic reference electrode fixed to the deltoid muscle predominantly involves cerebellar structures, mainly the cerebellar cortex. It has been shown that effects are hardly influenced by fine placement of the stimulating electrode but somehow by an individual anatomical variability. Moreover, similar to the use of transcranial magnetic stimulation (TMS) a varying individual sensitivity to non-invasive brain stimulation has been proposed also in the application of tDCS as revealed by modulated amplitudes of motor evoked potentials (Labruna et al., 2016). Those individual components may also play a role in the efficacy of cerebellar tDCS on eyeblink conditioning and possibly other paradigms.

However, the critical role of the distance of the stimulating electrodes for the duration and magnitude of induced after-effects has been highlighted by Moliadze et al. (2010). Stimulating the primary motor cortex and using different montages of the reference electrode revealed evidence that the stimulation intensity has to be adapted in particular when extracephalic reference electrodes were placed at the ipsi- or contralateral arm. In the present study stimulation intensities were not adapted neither when the extracephalic nor the cephalic reference electrode was used.

The efficacy of tDCS on structures within the cerebellar cortex may also depend on the direction of the induced current. Current direction related effects have been reported using TMS over the cerebellum (Ugawa et al., 1994) and in various brain areas like the primary motor cortex and the visual cortex as revealed by thresholds for evoked responses (Kammer et al., 2001; Richter et al., 2013). There is probably no direct one-to-one translation between TMS coil orientation and tDCS current direction, however, a modification of the current flow may also influence tDCS after-effects (Moliadze et al., 2010). Although this explanation is largely hypothetical, future studies that include computational modeling of cerebellar tDCS and TMS techniques can shed light to this issue.

At further variance with our previous study, it has to be noted that tDCS was applied only within the first half of paired trials and not throughout the entire acquisition phase. However, distinct between group effects in the study by Zuchowski et al. (2014) were already observed with beginning of the stimulation period. In addition, intertrial intervals were shorter in the present study. Finally, larger individual differences in the ability to acquire eyeblink conditioning in subjects studied by Zuchowski et al. (2014) may have confounded previous data.

In addition to findings in CR acquisition timing of conditioned eyeblink responses in paired trials was not significantly affected by tDCS in the present experiments, differences between the stimulation groups were not significant. In our previous study, anodal stimulation was followed by significantly shortened CR onset latencies that is mean onset of responses was shifted closer to CS onset. This did not necessarily imply a less appropriate CR timing (Ebel and Prokasy, 1963; Millenson et al., 1977). Because the number of CRs was markedly reduced in the previous cathodal group, delayed CR onset was discussed as less reliable. However, in parallel to the lack of significant effects on CR acquisition timing of CRs was not altered in the present study and no clear conclusions can be drawn on timing by the present data.

In both of the first two experiments with stimulation during acquisition participants showed a decline of CR rates during extinction trials, no significant group differences were observed. To study effects of tDCS on extinction more directly, in the third experiment tDCS was given only during extinction trials using the cephalic reference electrode. Repeated presentation of the CS alone was followed by a decline of CRs as observed already in the first extinction block in each stimulation group. No significant differences occurred neither on extinction effects nor the amount of savings on the next day. As suggested by animal data, extinction is in part an active process distinct from acquisition to unlearn previously learned behavior (Robleto et al., 2004; Hu et al., 2015). Moreover, acquisition is thought to involve initially the cerebellar cortex, then the cerebellar nuclei. After extinction plasticity remains in the cerebellar nuclei, but not the cerebellar cortex and cerebellar nuclei have been proposed to contribute to savings ( for review, Medina et al., 2001; Mauk et al., 2014). To date, few human lesion studies investigated the contribution of the human cerebellum to CR extinction (Timmann et al., 2005; Gerwig et al., 2006, 2010; Ernst et al., 2016). In short, because of significantly reduced acquisition in cerebellar patients, no clear conclusions on extinction or savings could be drawn. Brain imaging studies in healthy subjects, however, provide support that the human cerebellum contributes to extinction (Parker et al., 2012; Thürling et al., 2015). In addition to lesion and imaging studies there are findings from cerebellar stimulation on extinction and retention. In our previous tDCS study the initial extinction rate appeared to be faster following anodal tDCS. It has to be noted that tDCS was given only throughout the acquisition phase and that anodal stimulation led to higher mean CR incidences. Across the three extinction blocks, however, there was no significant difference comparing anodal, cathodal and sham stimulation. Effects on savings were not assessed (Zuchowski et al., 2014). In the present study relatively low mean CR incidences in paired trials may have contributed to the lack of significant group effects in extinction, in addition, the duration of tDCS may have been too short in extinction trials to evolve efficacy. It is a matter of debate whether the main effect of stimulation may affect the cerebellar cortex. In a study by Monaco et al. (2014) inhibitory cerebellar continuous theta-burst stimulation (cTBS) after a first learning session and was found to impair extinction as part of a fast adaptation process. In agreement with another cTBS study (Hoffland et al., 2012) reacquisition was not disturbed. It has been concluded that stimulation may have altered plasticity in the cerebellar cortex involved in extinction, but not plasticity in the deep cerebellar nuclei involved in savings. Findings of unaffected savings in the present study may be consistent with that. Because we were interested in effects on long lasting consolidation processes in eyeblink conditioning and less in tDCS after effects lasting for hours, retention was tested on the next day. At variance with that Monaco et al. (2014) retested their patients 1 week apart.

The aspects discussed above, especially the different mode to apply tDCS represent clear limitations to compare findings of the present study with our previous data. Replication of previous findings needs to be addressed in further studies since a complex interaction between several factors of the application of cerebellar tDCS and eyeblink conditioning is suggested. Investigating few participants in each group, the present data are further limited by a low statistical power. It would have been useful to replicate the exact same methodology of our previous study (Zuchowski et al., 2014) and to compare results with the present data, especially with Experiment 1 and 2.

## Conclusion

In the present study polarity dependent effects of cerebellar tDCS on the acquisition and timing of conditioned eyeblink responses were not confirmed as previously reported. In addition, cerebellar tDCS during extinction trials did not modulate extinction and reacquisition. Several aspects may explain the weaker effects like the beginning of tDCS before the learning phase and individual factors influencing current flow and thresholds. Future studies in larger subject populations should address parameters of stimulation and eyeblink conditioning paradigms which lead to robust cerebellar tDCS effects.

## Author Contributions

LB, DT and MG: substantial contributions to the conception or design of the work, the acquisition, analysis and interpretation of data for the work. LB, GB, DT and MG: drafting the work or revising it critically for important intellectual content; agreement to be accountable for all aspects of the work in ensuring that questions related to the accuracy or integrity of any part of the work are appropriately investigated and resolved. GB, DT and MG: final approval of the version to be published.

## Financial Disclosures

LB and GB reports no disclosures. DT received research support from the German Research Foundation (DFG). MG received speaker honoraria and/or travel reimbursement from Novartis, Pfizer and Ipsen Pharma and research support from MSD.

## Conflict of Interest Statement

The authors declare that the research was conducted in the absence of any commercial or financial relationships that could be construed as a potential conflict of interest.
